# Comparative analysis of 4C-Seq data generated from enzyme-based and sonication-based methods

**DOI:** 10.1186/1471-2164-14-345

**Published:** 2013-05-24

**Authors:** Fan Gao, Zong Wei, Wange Lu, Kai Wang

**Affiliations:** 1 Eli and Edythe Broad Center for Regenerative Medicine and Stem Cell Research, Department of Biochemistry and Molecular Biology, University of Southern California, Los Angeles, CA 90089, USA; 2 Zilkha Neurogenetic Institute, Department of Psychiatry and Department of Preventive Medicine, University of Southern California, Los Angeles, CA 90089, USA

**Keywords:** Circular chromosome conformation capture, Sonication, 4C-Seq, Bioinformatics, Next-generation sequencing

## Abstract

**Background:**

Circular chromosome conformation capture, when coupled with next-generation sequencing (4C-Seq), can be used to identify genome-wide interaction of a given locus (a “bait” sequence) with all of its interacting partners. Conventional 4C approaches used restriction enzyme digestion to fragment chromatin, and recently sonication approach was also applied for this purpose. However, bioinformatics pipelines for analyzing sonication-based 4C-Seq data are not well developed. In addition, data consistency as well as similarity between the two methods has not been explored previously. Here we present a comparative analysis of 4C-Seq data generated by both methods, using an enhancer element of *Pou5f1* gene in mouse embryonic stem (ES) cells.

**Results:**

From biological replicates, we found good correlation (r>0.6) for inter-chromosomal interactions identified in either enzyme or sonication method. Compared to enzyme approach, sonication method generated less distal intra-chromosomal interactions, possibly due to the difference in chromatin fragmentation. From all mapped interactions, we further applied statistical models to identify enriched interacting regions. Interestingly, data generated from the two methods showed 30% overlap of the reproducible interacting regions. The interacting sites in the reproducible regions from both methods are similarly enriched with active histone marks. In addition, the interacting sites identified from sonication-based data are enriched with ChIP-Seq signals of transcription factors Oct4, Klf4, Esrrb, Tcfcp2i1, and Zfx that are critical for reprogramming and pluripotency.

**Conclusions:**

Both enzyme-based and sonication-based 4C-Seq methods are valuable tools to explore long-range chromosomal interactions. Due to the nature of sonication-based method, correlation analysis of the 4C interactions with transcription factor binding should be more straightforward.

## Background

Chromosomal organization in the nucleus has been gradually recognized as an important contributor to gene regulation and genome function. In the last decade, chromosome conformation capture (3C) has been developed as a high-throughput assay to explore long-range chromatin-chromatin interactions *in vivo*[1]. Technologies derived from 3C, such as Hi-C [2,3], 5C [4] and TCC [5], explore global chromatin interactions. Other methods like ChIP-3C [6-10] and ChIA-PET (chromatin interaction analysis by paired-end tag sequencing) detect chromatin-chromatin interactions mediated by a specific DNA binding protein [11-13]. A particular derivative of 3C method named circular chromosome conformation capture (4C) enables *de novo* detection of all interacting partners of a known genomic region, such as differentially methylated H19 imprinting control region [14]. Similar to 3C-based global mapping of chromatin-chromatin interactions, 4C follows the same concept of 1) crosslinking of protein-DNA complexes in the cell nucleus to capture chromosome conformation and 2) proximity ligation of physically interacting chromatin fragments. However, instead of using immuno-precipitation to capture the ligated junction DNA pieces in ChIP-3C and ChIA-PET, 4C utilizes PCR reactions to enrich genomic regions interacting with a known “bait” region. The PCR products generated from the 4C technology can be subsequently examined by different approaches: they can be cloned for Sanger sequencing [15], or hybridized to a microarray [14,16]. Given the rapid development of next-generation sequencing, it is now feasible to directly interrogate the PCR products from the 4C technology (hereafter referred to as “4C-Seq”). This technique enables genome-wide mapping of the chromatins interacting with the “bait” in greater resolution and precision.

In most of the published 4C studies to date [14,16-20], 4C library preparation typically starts with formaldehyde crosslinking of chromosomes *in vivo*, followed by restriction enzyme digestion to fragment chromosomes. After proximity ligation in diluted condition, chromatin proteins are digested with proteinase K and formaldehyde crosslinks are reversed. Finally “bait”-containing circular DNA molecules generated from the ligation are amplified using bait-specific primers in nested PCR reactions. Although enzyme digestion-based 4C method has limitations such as noisy/weak chromatin associations [21,22], low chromatin accessibility-dependent digestion efficiency as well as uneven distribution of restriction digestion sites across the genome, it should be acknowledged that recent advance in enzyme-based 4C method greatly increased data resolution and robustness by using 4-bp cutter instead of 6-bp cutter in fragmentation [23]. Compared to enzyme digestion, sonication is less accessibility-dependent and preferentially breaks crosslinked chromosomes at the edge of protein binding sites [24,25]. Thus sonication-based approach provides an alternative choice for 4C-Seq studies [26], which is potentially more straightforward on exploring bound transcription factor(s) that mediate the interactome.

Unlike enzyme digestion-based 4C method [17-19], sonication-based library presents a data analysis challenge which requires a different set of analytical approaches. The major reason is that each restriction enzyme has a set of known breakage sites in the genome, so that researchers typically align all reads against flanking sequences of this set of restriction enzyme site, and the restriction sites are the exact ligation sites between two interacting regions [18]. In contrast, sonication method has generally no preference of sequence motifs at breakage sites. Without knowing the exact ligation sites a priori, the pipeline for data processing is anticipated to be different from enzyme digestion-based method. So far, the bioinformatics pipelines have not been well developed to process sonication-based 4C data generated by high-throughput short-read sequencers, such as Illumina Hi-Seq. In the current study, we describe a sonication-based 4C-Seq protocol that we applied to explore *Pou5f1* enhancer interactome in mouse ES cells. The *Pou5f1* enhancer has been known to mediate expression of its own gene product – Oct4, a key reprogramming factor [27]. Thus exploration of this enhancer interactome will provide better understanding of *Pou5f1*-related regulatory network. To compare different 4C protocols, we also performed a parallel study using an enzyme-based method described in [18]. We analyzed consistency of processed 4C-Seq data generated from biological replicates, applied statistical analysis to identify enriched interacting regions, compared the reproducible enriched interacting regions identified from enzyme and sonication methods, and explored epigenetic features enriched in the 4C interactome.

## Results and discussion

### Overview of sonication-based 4C library preparation

**Figure 1:**
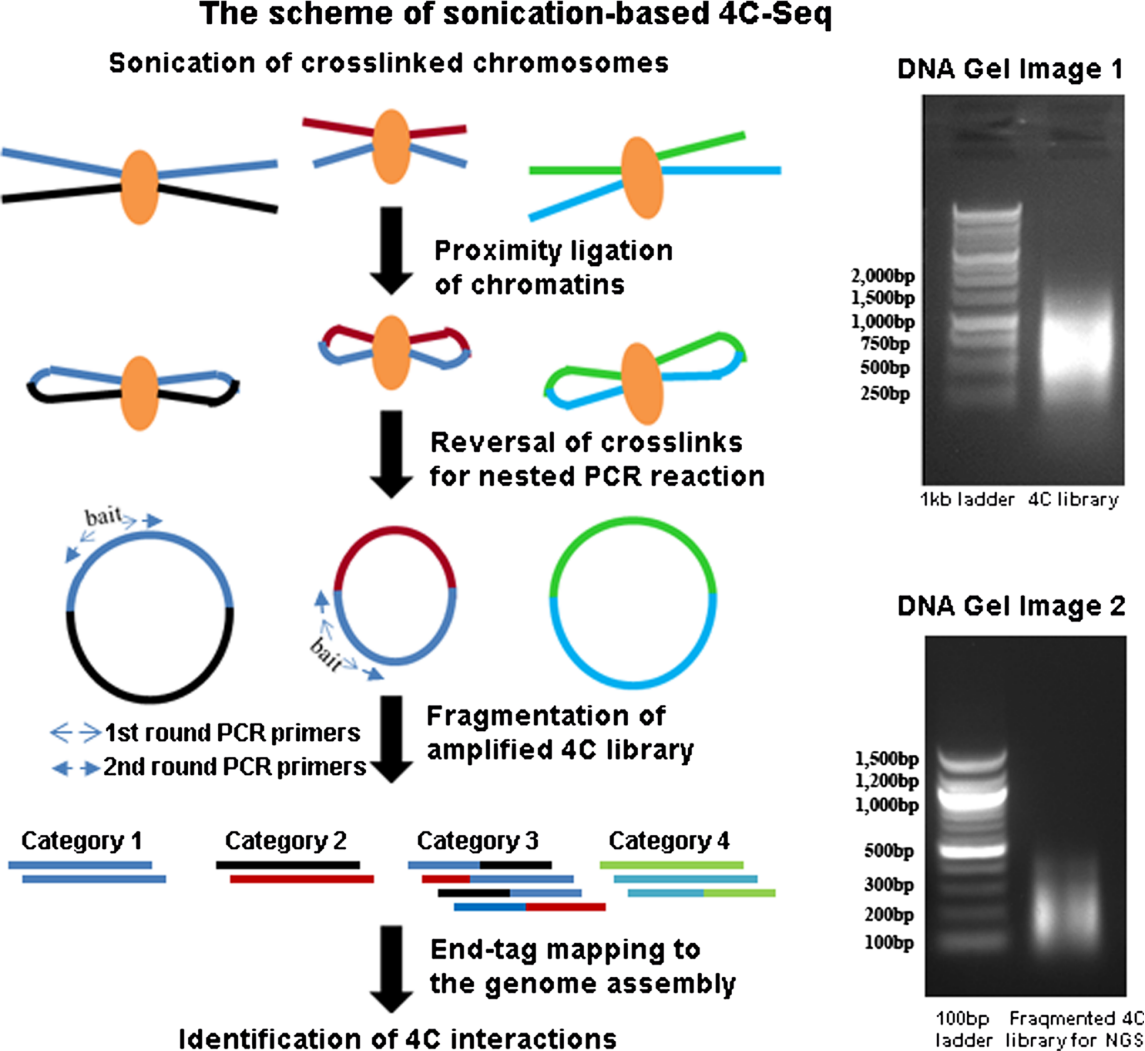
**The procedure of sonication based 4C-Seq. A**) The scheme of 4C-Seq process to generate high-throughput sequencing data for analysis. Two rounds of PCR reactions (nested PCR) were used to amplify bait interacting regions (primers shown in arrows). The fragmented DNA pieces for sequencing can be classified in four categories shown in the left panel. **B**) DNA electrophoresis gel images of constructed 4C library from nested PCR. **C**) DNA electrophoresis gel images of 4C library after fragmentation for NGS.

Based on previously published 4C studies on KRT gene cluster [26], we slightly modified the experimental protocol for sonication-based 4C library preparation (Figure 1, see Methods for details). We chose an upstream enhancer element of *Pou5f1* gene in mouse ES cells as bait and constructed 4C libraries for two biological replicate samples BR1 and BR2. Compared to the study by Huang et al. [26], our method on post-processing of the generated 4C library for next-generation sequencing is notably different. Huang et al. analyzed the amplified 4C products consisting of bait-target-bait DNA pieces using 454 Titanium Sequencer. In our study, to adapt to Illumina short-read sequencing, an additional sonication step fragments 4C products into ~200 bp DNA pieces containing bait, targets, bait-target junctions as well as genomic contaminants, before subject to Illumina Hi-Seq sequencing. Sonication of ligated DNA fragments to smaller pieces for Hi-Seq sequencing was also applied in a recent Hi-C study [28]. We used an end-tag mapping method, similar to the one described in [24] for identifying junction reads from sonication-based 4C-Seq data. In our study, enzyme-based 4C-Seq library preparation strictly followed the protocol described in [18].

### Data processing framework

Based on our experimental protocol, the short sequencing reads from Illumina Hi-Seq platform should theoretically fall into four categories: 1) reads at the 4C bait locus, 2) reads at the bait interacting regions, 3) reads that spans ligation junctions between bait locus and its interacting region, as well as 4) noises caused by contamination of genomic or circular DNA that are not amplified in PCR reactions (Figure 1A). We therefore evaluated different strategies to identify these four types of reads.

We first attempted to separately map both forward and reverse reads (91 bp) of the paired-end data to the reference genome (mm9) using Burrows-Wheeler Aligner (BWA [29]), to identify the reads that are completely aligned to the “bait” locus or other genomic regions. For sequencing data generated from the two biological replicates, the mapped reads account for ~77% of the total reads (Additional file 1: Table S1), with most of the mapped reads being uniquely mapped. With this mapping strategy, >99% of the uniquely mapped reads are within the bait locus, suggesting the presence of many proximal interactions or self-ligations in the data. Only 0.2% to 0.6% of the uniquely mapped reads are mapped to distal genomic regions, that is, the majority of them should fall into Category 2 described above, though it is possible that some of them may originate from genomic DNA described in Category 4. We also note that unmapped reads in the biological replicates account for ~23% of total reads, which may correspond to ligation junctions that cannot be mapped to the reference genome, that is, Category 3 above. Given the limited amount of data supporting Category 2, we believe that this mapping strategy is not optimal for identifying bait interacting regions.

We next evaluated an end-tag mapping strategy [2,11], generally applied in 3C-based studies to identify Category 3 reads that are mosaic of the bait and its interacting regions. A similar strategy was also previously used in a ChIA-PET study [30], with 20-bp end tags. Here we define “bait region” as a ~1 kb region, which includes 500 bp extension from the locations of the 2nd set of forward and reverse PCR primers (Figure 1). We extracted 20-bp end tags from both forward and reverse sequencing reads and aligned them to the reference genome assembly separately using BWA [29]. The generated forward and reverse alignment files were merged together using SAMtools [31]. Junction reads are identified, when one end-tag uniquely maps to the “bait” and the other end-tag maps to genomic locations > 300 bp away on the same chromosome (intra-chromosomal interactions) or to a different chromosome (inter-chromosomal interactions). The rationale to choose 300 bp is that our sonication approach generates small DNA pieces with an average size of 200 bp for sequencing, so end-tags that are > 300 bp away should mostly be junction reads. We next classified junction reads as proximal junction reads and distal junction reads. Proximal junction reads have two end tags mapped on the same chromosome with genomic distance between the tags between 300 bp and 10 kb. Distal junction reads are either two tags on the same chromosome with distance greater than 10 kb or two tags on different chromosomes. Proximal junction reads account for ~90% of total junction reads identified (Additional file 1: Table S2), with the distribution of relative genomic distance between the two ends following a continuous decay, starting from 300 bp to 2 kb, similar to the self-ligation events observed in a ChIA-PET study [30]. Predominant proximal ligation may reflect disruption of weak chromatin-chromatin interactions under the shearing force, thus facilitating self-ligation events. Since no interactions were identified within the distance range from 2 kb to 10 kb in both BR1 and BR2 biological replicate data, we used 10 kb distance cutoff to distinguish proximal *vs.* distal intra-chromosomal interactions.

To explore distal chromatin-chromatin interactions, we processed distal junction reads for further analysis. Tags that were within 100 bp range on their genomic locations were considered as PCR products from a single ligation event and merged as one unique distal interacting site, given that the 4C library DNA was fragmented before sequencing. Unique distal interacting sites supported by only one read were removed from our analysis as they likely represent background noises. In total, using 20 bp end-tag mapping, we identified 5,705 and 4,368 filtered unique interacting sites respectively from two biological replicate data generated from sonication-based 4C method. In the following sections, we will discuss 4C sequencing data in the context of data reproducibility, effect of sequencing depth, statistical models for identifying enriched interacting regions, comparison of reproducible interacting regions identified in both enzyme and sonication methods, epigenetic histone features surrounding the interacting sites within the reproducible regions, and transcription factors enriched around sonication generated interacting sites.

### Reproducibility of the inter-chromosomal interactions

**Figure 2:**
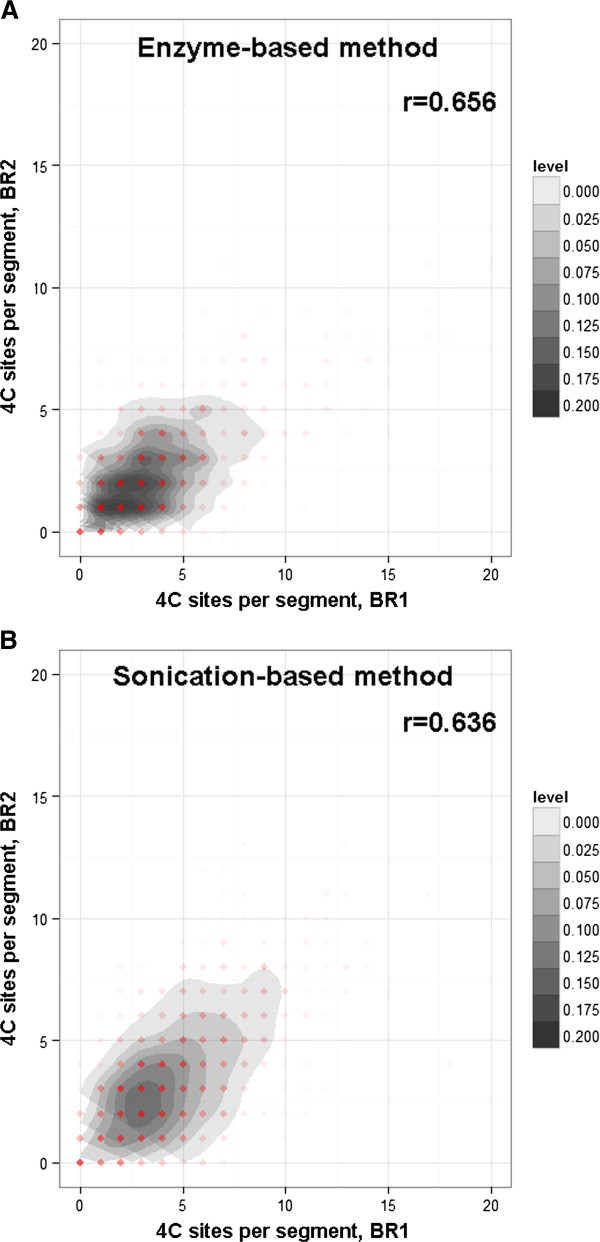
**Reproducibility of inter-chromosomal interactions.** Density scatter plots of inter-chromosomal interactions identified in the biological replicate data for both sonication-based and enzyme-based methods. Pearson’s correlation coefficient values were also shown in the upper right corner of the plots.

Chromatin-chromatin interactions are highly dynamic [32], and the interactions are probably more transient than protein-chromatin interactions in the nucleus. For example, CTCF mediated interactome in mouse ES cells has only 38% overlap between biological replicates, suggesting dynamic feature of CTCF mediated chromatin-chromatin interactions; while 98% peaks identified in CTCF ChIA-PET study can be found in ChIP-Seq peak data of CTCF, reflecting strong association of CTCF with chromatin fibers [12]. We note that the 4C-Seq approach aims to take a snapshot of chromatin interacting patterns, which reflect the average state across hundreds of thousands of cells. Genome-wide 3C/Hi-C, ChIA-PET studies revealed more consistent proximal interactions than distal interactions. It is likely that sequencing depth for such studies is not sufficient for capturing less frequent distal interactions, such as inter-chromosomal interactions. Compared to 3C-based studies, 4C-Seq explores the interactions associated with only one bait area, thus in theory, it enables a more thorough search of less frequent long-range interactions, provided the same sequencing depth of the prepared DNA libraries. Therefore, we decided to determine the reproducibility of inter-chromosomal interactions between biological replicate samples by counting the number of observed interactions in every genomic bin and calculate the correlation between replicates. The correlation of the data is an indication whether the bait region has preferred interacting partners located on different chromosomes or the inter-chromosomal interactions mainly result from random collision between the chromosomes. As for 4C-Seq data, the reproducibility can also be affected by many factors including efficiency of proximity ligation, PCR amplification, DNA fragmentation, the quality and depth of next-generation sequencing, as well as the data processing strategy. Theoretically, sticky-end ligation applied in enzyme-based method should result in higher ligation efficiency than blunt-end ligation in sonication-based method; however, smaller size of chromatin fragments generated by sonication may contribute to higher chance of collision frequency between two breakage points. In our analysis, the replicate interactome data of the *Pou5f1* enhancer generated from both methods were included for comparison (Additional file 2: Figure S1). The recommended data processing protocol for analyzing enzyme-based inter-chromosomal interactions [18] was used to count the number of ligated HindIII sites for each genomic segment that covers 500 HindIII sites. For 4C-Seq data generated by sonication method, we counted the number of identified unique interacting sites in each 2 Mb genomic bin (roughly the size covering 500 of 6-bp cutters) to explore the correlation between biological replicates. For inter-chromosomal interactions generated in biological replicate mouse ES cells, we found Pearson’s correlation coefficient values of 0.658 and 0.636 for enzyme-based and sonication-based 4C libraries respectively (Figure 2). When we attempted to use smaller bin sizes (1 Mb and 500 kb) for sonication-based data, the correlation coefficient between the replicates decreased to 0.559 and 0.473 respectively (Additional file 3: Figure S2A&C). For enzyme-based data, using smaller bin sizes (250 and 125 6-bp sites) also resulted in a similar decrease of correlation coefficient values (r=0.581, r=0.475; Additional file 3: Figure S2B& D). Thus, the two methods showed similar reproducibility, and sonication-based method doesn’t improve or degrade the resolution in this case. Also in our experiments, the biological replicate data for enzyme-based 4C libraries showed that 36% of the inter-chromosomal interactions identified in the second replicate are in close proximity (within 10 kb range) to the interactions in the first replicate, whereas 46% of the inter-chromosomal interactions from sonication-based 4C libraries showed proximity between biological replicate. This analysis indicated that interacting regions identified from biological replicates are relatively consistent with each other, in spite of different methods applied in chromatin fragmentation.

### Evaluation of sequencing depth

**Figure 3:**
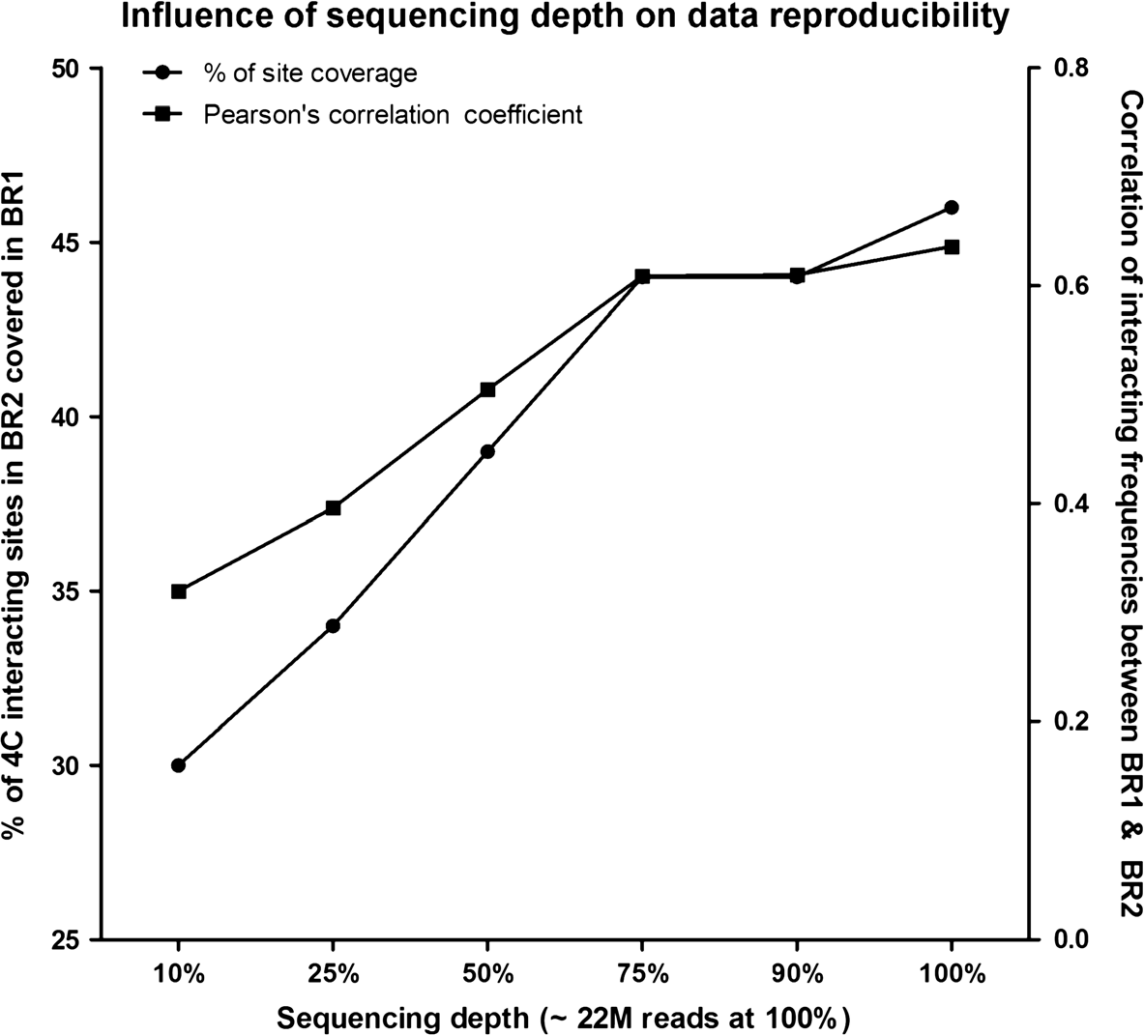
**Sequencing depth dependent data reproducibility.** Overage of interacting sites and Pearson’s correlation coefficient of domain interacting frequencies were plotted separately for sonication-based 4C data.

Intuitively, sequencing depth is directly related to the ability to find relatively rare interacting events, as well as the true fraction of the reads that can be informative to finding distal interacting events. We explored with 10%, 25%, 50%, 75%, 90% and 100% of the original sequencing data to perform the same set of analysis for the replicate data of sonication-based 4C libraries, and analyzed their correlation patterns. As shown in Figure 3, with sequencing depth rose from 10% to 100%, the coverage of identified clustered distal interacting sites in the two biological replicates (BR1 and BR2) gradually increased from 30% to 46% (coverage defined as the percentage of BR2 sites within 10 kb of BR1 sites). More importantly, pairwise correlation of interacting frequencies of 2 Mb genomic bins between BR1 and BR2 showed an increment from 0.328 to 0.636. However, when more than 75% of the sequencing reads were used in analysis, both coverage of the interacting sites and correlation of the interacting frequencies in the two replicates reached a plateau. Therefore, ~20 million total short reads (10 million read pairs) from Illumina sequencing is sufficient to capture most of the interacting events of this enhancer element in mouse ES cells.

### Statistical analysis to identify enriched interacting regions from the inter-chromosomal interactions

As a high-throughput assay, 4C-Seq revealed thousands of sites interacting with one bait region. However, it is unlikely that all the interactions identified are biologically significant, and many of them probably represent random collision between two genomic fragments in 3D space. To identify regions that are frequently associated with the bait region other than random collision, we applied statistical models to analyze interacting sites within each chromosome. We used a permutation-based false discovery rate (FDR) procedure to choose significantly enriched interacting regions. For enzyme-based 4C data, a z-score was assigned based on the number of 4C interacting sites per 500 HindIII sites [18]. For sonication-based data, each interacting site was assigned a z-score based on the nearby interactions observed within ±1 Mb distance range (see Methods for details). FDR was calculated by random permutation of the data 100 times, and the cutoff value of 5% was used to select positive sites. Positive sites and the nearby interaction sites (±1 Mb range) were grouped together as enriched interacting regions. Overlapping enriched regions were further merged together. The statistical models applied here aim to identify enriched interacting regions from the background, similar to the concept used in ChIP-Seq peak calling.

### Comparison of enriched interacting regions identified from 4C-Seq data generated from different methods

**Figure 4:**
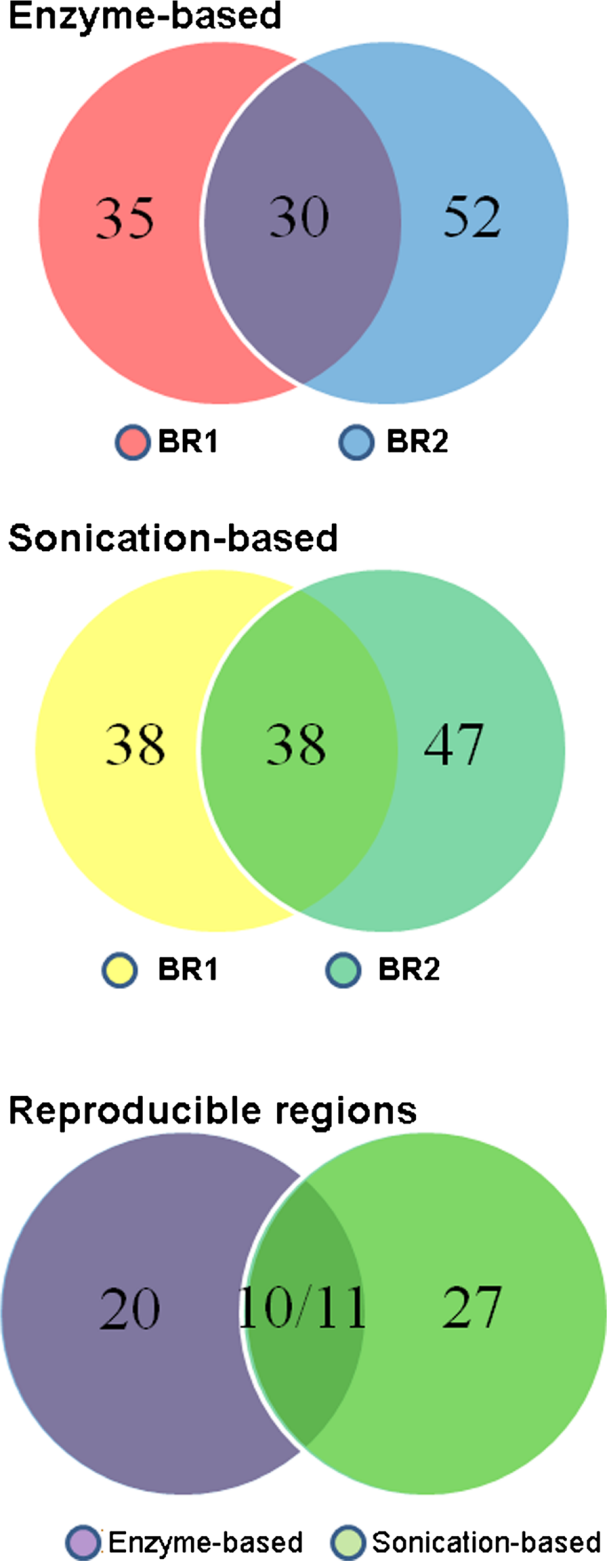
**Overlap of inter-chromosomally enriched interacting regions. A**,**B**) Overlap of enriched inter-chromosomal interacting regions identified in the biological replicate data generated using enzyme-based or sonication-based 4C-Seq approaches; **C**) Overlap of reproducible inter-chromosomal interacting regions between the two different 4C-Seq methods.

Statistical analysis identified 65 and 82 enriched inter-chromosomal interacting regions for each replicate data generated from the enzyme approach, with about 40% overlap of the identified regions (30 regions) between the two biological replicates (Figure 4A). For sonication-based data, 76 and 85 regions were identified from each replicate, with nearly 50% of the regions are overlapping between the two replicates (Figure 4B). Thus those reproducible regions represent high-confidence interactions that might have biological consequences. It is interesting to note that one third of the reproducible inter-chromosomal regions identified in the enzyme method overlap with the reproducible regions from sonication-based data (Figure 4C). Thus, the *Pou5f1* enhancer shows preference in interacting with distal regions that are located on different chromosomes, and the identified reproducible regions even possess certain level of consistency between sonication-based and enzyme-based 4C-Seq approaches.

### Comparison of distal intra-chromosomal interactions

**Figure 5:**
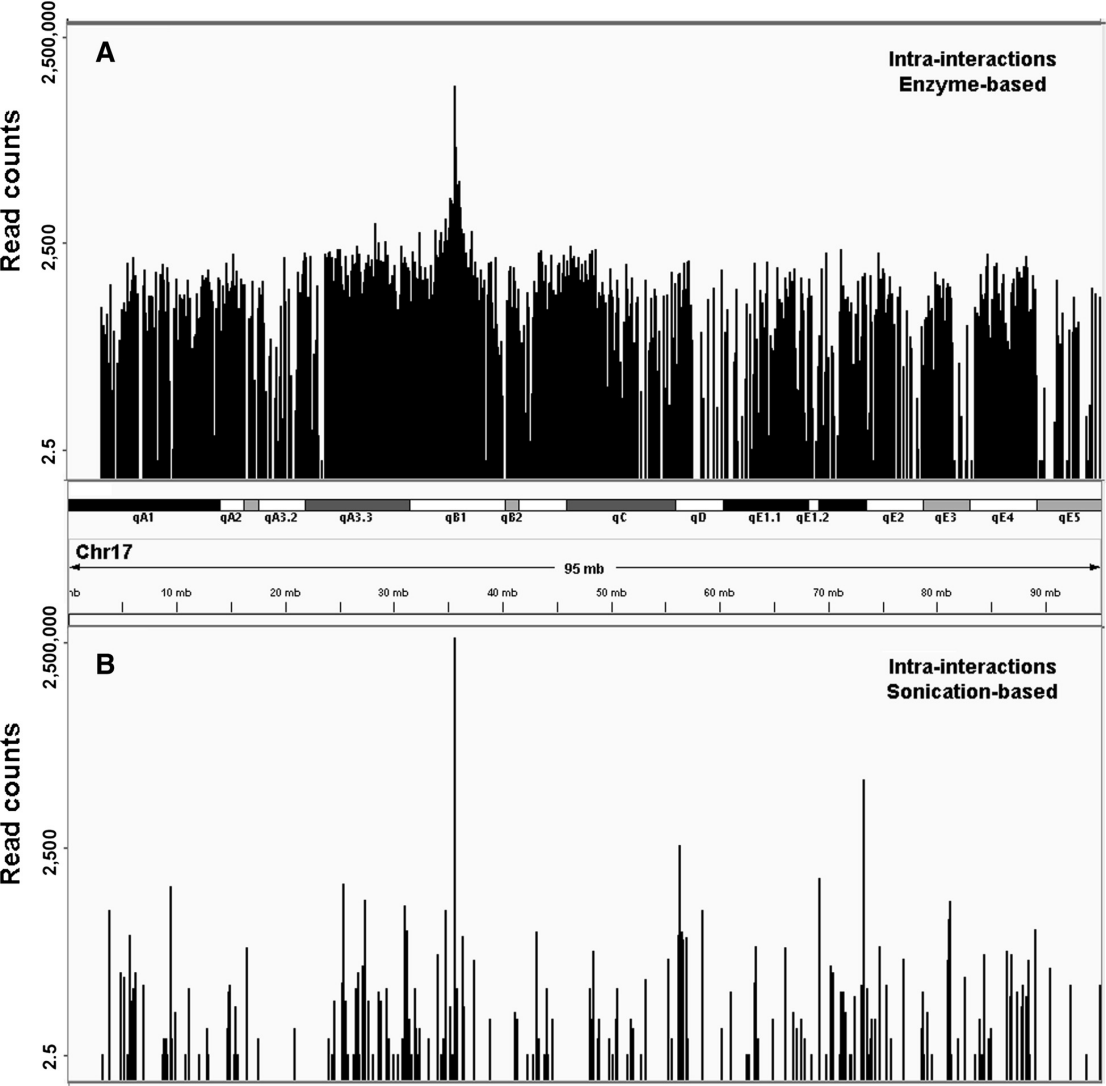
**Distribution of raw read counts at all intra-chromosomal interacting sites. A**) Distribution of read counts at *cis*-interacting sites identified from enzyme-based method; **B**) Distribution of read counts at *cis*-interacting sites in sonication-based data.

Intra-chromosomal interactions include both proximal *cis*-interactions around the bait locus and long-range *cis*-interactions distal to the bait area. Both enzyme and sonication-based 4C-Seq data revealed a majority of proximal *cis*-interactions in all the interaction reads identified (Additional file 1: Table S2 & S3). As shown in the distribution plot for the identified intra-chromosomal interaction reads (Figure 5), distal interactions occur even at 60 Mb away from the bait location. Interesting to note, sonication-based method generated less distal intra-chromosomal interactions compared to enzyme-based 4C-Seq method (Figure 5). For enzyme-based 4C-Seq, 18.5% and 12.6% of all the HindIII sites on Chromosome 17 were identified as interaction sites for the two biological replicate data respectively, suggesting a high background (Figure 5B) in intra-chromosomal interactions, similar to the observations published by de Latt group [18]. Data generated by sonication-based method is consistent with the 4C-Seq data from Ruan group with only a few distal intra-chromosomal interactions [11]. Clearly, in our case, we did not observe predominant distal intra-chromosomal interactions within all the distal interactions from sonication-based method, contrasting to the data generated from the enzyme method we used. Sequencing depth in general, affects interactions identified; however, since we are examining ratio of distal intra-chromosomal interactions among all interactions, differences in sequence coverage is unlikely to play a major role. Interesting to note, a previous e4C study [33] showed predominant inter-chromosomal interactions of *Hbb* locus by using an array-based 4C technique. We suspect that sonication-based method may have shaken off a lot of weak interactions [22] that were identified in the enzyme-based approach we applied.

**Figure 6:**
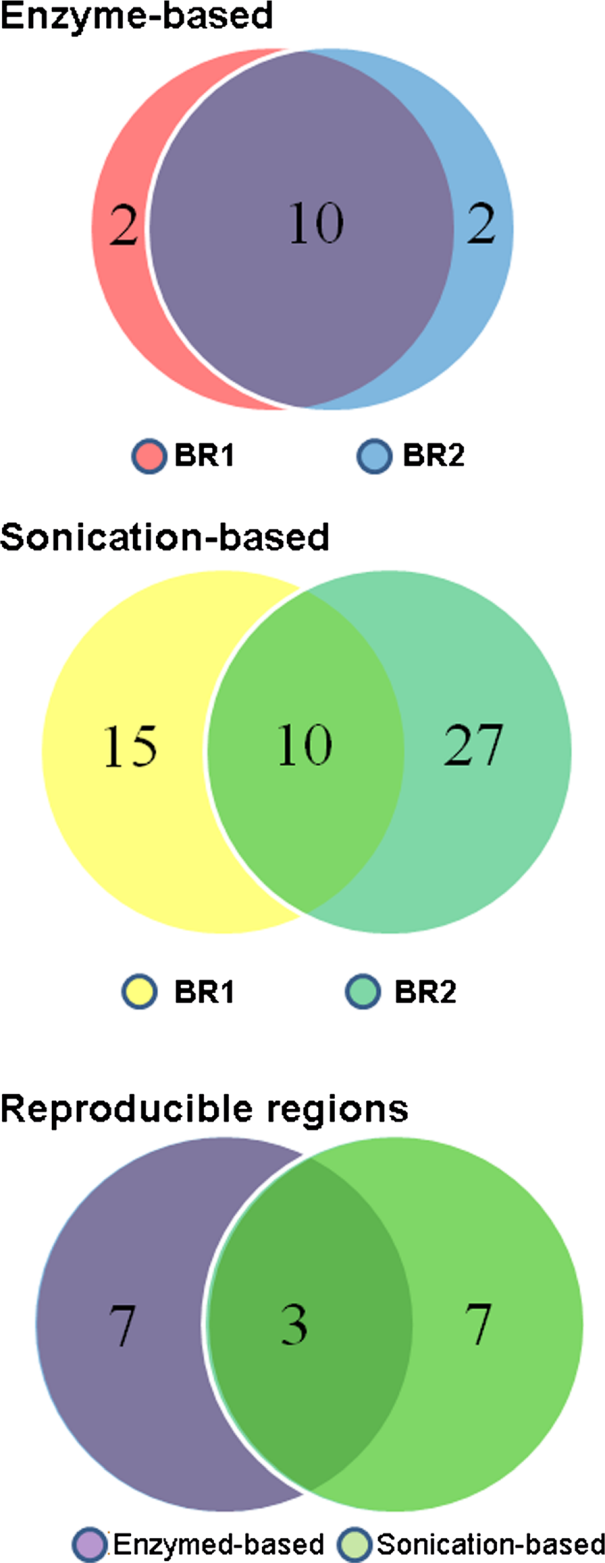
**Overlap of intra-chromosomally enriched interacting regions. A**,**B**) Overlap of enriched intra-chromosomal interacting regions identified in biological replicate data generated using enzyme-based or sonication-based 4C-Seq approaches; **C**) Overlap of reproducible distal intra-chromosomal interacting regions from enzyme-based and sonication-based 4C-Seq approaches.

For enzyme-based 4C-Seq data, we followed a statistical model [18] to identify enriched *cis*-interacting regions. In brief, z-scores in windows covering 100 enzyme sites (size ~400 kb) were calculated based on contact frequencies in each window, with a background window covering 3,000 sites to calculate expected contact frequency. A permutation-based FDR method with a threshold (FDR≤ 5%) was used to select enriched-interacting regions as described above for the inter-chromosomal interactions. Shown in Figure 6, the coverage between the biological replicate data is above 80%. For sonication-based method, windows of ± 200 kb around identified contact sites were used to identify enriched-interacting regions (see methods). The overlap of enriched-interacting regions between the two replicate data is ~33%, lower than enzyme-based method. Still, 30% of the reproducible regions overlapped between the two methods.

### Epigenetic histone marks enriched in the interactomes

**Figure 7:**
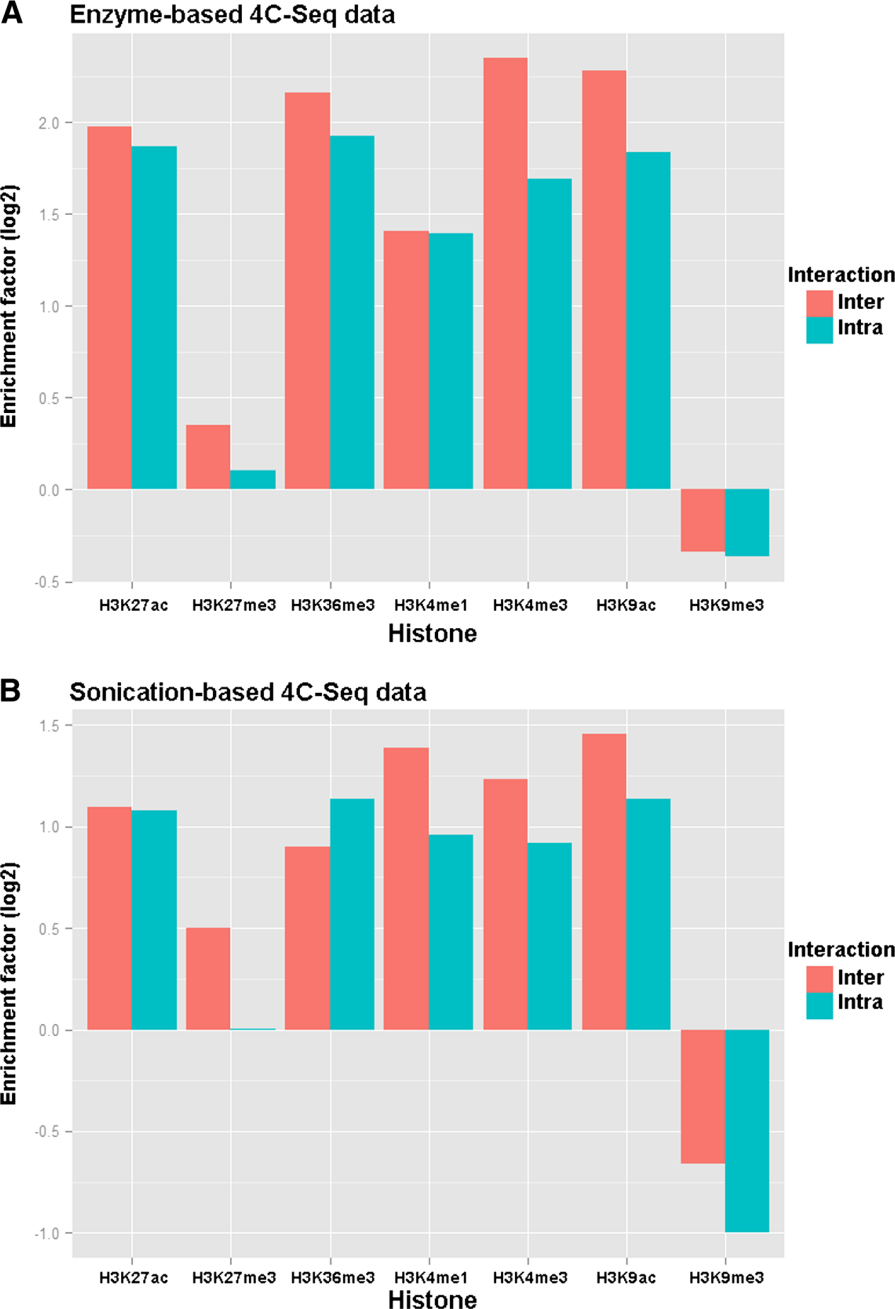
**Enrichment analysis of histone marks.** Bar plots of enrichment factor values of different histone marks around the interacting sites within the reproducible interacting regions. Enrichment factor was calculated as the observed sites in proximity to the ChIP-Seq peaks of a particular histone mark divided by the expected sites (random simulated across chromosome 17) close to that mark.

Previous Hi-C study unveiled chromosomal organization of open and closed chromatin compartments in the cell nucleus [2], with open chromatin compartments enriched with active epigenetic features. We questioned whether the interactomes of active *Pou5f1* enhancer are associated with specific epigenetic features. Thus we performed association study to calculate enrichment factors for a series of histone marks in the interactomes. For both enzyme-based and sonication-based data, histone marks related to gene activation, such as H3K27ac, H3K36me3, H3K4me1, H3K4me3 and H3K9ac were enriched around the identified contact sites (±5 kb range) within the reproducible interacting regions (Figure 7). In contrast, enrichment for H3K27me3 repressor mark and H3K9me3 heterochromatin mark was either not obvious or not observed at all. Thus in our study, 4C-Seq data revealed physical proximity of an enhancer element with distally active genomic regions in mouse ES cells, consistent with the concept of active genomic compartments from genome-wide 3C studies [2,5]. These results therefore serve as positive controls for our experiments, and confirmed that biological insights can be inferred from both enzyme-based and sonication-based 4C-Seq data sets with appropriate analytical approaches.

### Transcription factors enriched in the interactomes

**Figure 8:**
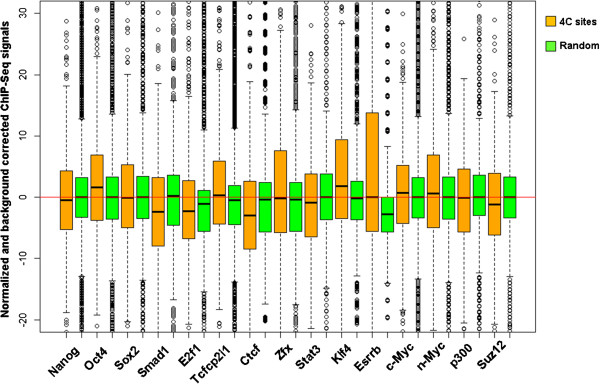
**Enrichment analysis of DNA-binding proteins.** Box plots showing distribution of normalized and background subtracted ChIP-Seq tag density of 15 DNA-binding proteins in mouse ES cells. Comparison was made between the 4C interacting sites (brown colored) and random iterated sites (green colored).

Unlike enzyme-based method, sonication-based 4C method applied ultrasound to shear cross-linked chromosomes into smaller pieces, similar to the fragmentation step in ChIP-Seq protocol. If particular DNA-binding protein complexes are involved in mediating chromosomal interactions between the bait and other distal regions, the identified bait-interacting sites from sonication-based data should be in close proximity to the bound sites of those DNA-binding proteins. We analyzed ChIP-Seq raw read files of 15 DNA-binding proteins reported in Chen et al’s study [34]. Briefly, the read tags within ±1 kb range of a 4C site was counted and normalized as having 10 million total read tags. The input data for the study was used to generate normalized background read counts. Specific ChIP-Seq read counts were calculated as background subtracted and plotted (Figure 8). Compared to randomly iterated genomic sites, the 4C interacting sites were moderately enriched with several transcription factors. Among them, transcription factors Oct4, Klf4, Esrrb, Tcfcp2i1 and Zfx showed statistically significant enrichment (p < 1×10^-10^, unpaired Wilcoxon-Mann–Whitney test), implying that these key pluripotency genes are mediating this enhancer interactome in mouse ES cells.

## Conclusions

In summary, we presented 4C-Seq data of an enhancer element in mouse ES cells, generated by two different methods: enzyme-based method and sonication-based method. Our data showed consistency of the data generated by both methods for biological replicate samples. For inter-chromosomal interactions, both methods have similar reproducibility of enriched-interacting regions; however, for intra-chromosomal interactions, enzyme-based method showed more frequent distal interactions and higher reproducibility. Both methods revealed that histone modifications related to gene activation were enriched in the interactomes. In addition, sonication-based data uncovered several key pluripotency genes enriched around the interaction sites. Thus, we conclude that 1) Both enzyme-based and sonication-based 4C-Seq technique are very useful tools for mapping long-range chromatin-chromatin interactions; and 2) 4C-Seq data together with ChIP-Seq data can help us elucidate molecular events surrounding a particular regulatory region in 3D space.

## Methods

### Cell culture and 4C library preparation

Mouse embryonic stem (ES) cell line E14 was grown in the culture dishes coated with 0.1% gelatin. A growth medium with Glasgow Minimum Essential Medium (GMEM) supplemented with 15% fetal bovine serum (FBS), 100 nM nonessential amino acids, 1% sodium pyruvate, 200 mM glutamate, 1% penicillin streptomycin, 50 uM b-mercaptoethanol and 10 ng/mL LIF was replaced every 24 hours to support ES cell growth. Immediately before 4C library preparation, 10 million cells were cross-linked with 1% fresh formaldehyde in the cell culture dishes, lifted and treated with Triton X100 buffer (0.25% Triton X100, 10 mM EDTA pH=8.0, 10 mM Tris–HCl pH=8.0, 100 mM NaCl, 1x protease inhibitor cocktail) to extract chromatins.

For sonication-based 4C library preparation, the isolated chromatin pellets were re-suspended in SDS lysis buffer (1% SDS, 5 mM EDTA pH=8.0, 50 mM Tris–HCl pH=8.0, 1x protease inhibitor cocktail) and sonicated to an average size of 500 bp. The diluted chromatin fragments were blunt-end repaired and ligated with T4 ligase for 24 hours at 4°C, followed by reverse crosslinking at 65°C for 20 hours with proteinase K. The purified DNA served as PCR template for 4C library construction. After nested PCR amplification for library construction, the purified PCR products (majority with size ranging from 500 bp to 1 kb) were further sonicated to small DNA fragments with an average size of 200 bp for sequencing. The Illumina HiSeq2000 Sequencer was used to perform paired-end sequencing with 90 bp read length.

For enzyme-based 4C library preparation, two rounds of enzyme digestions were carried out using HindIII and DpnII restriction enzymes respectively. The experimental procedure strictly followed the published protocol [35]. The bar-coded DNA libraries were subject to single-end sequencing with 50 bp read length using the Illumina HiSeq2000.

### Processing of 4C-Seq data

For sonication-based 4C-Seq data, Burrows-Wheeler Aligner (BWA) [29] with the “samse” option was employed for aligning paired-end data separately to the reference genome assembly mm9. Alignment files for both ends were further merged using SAMtools [31], followed by a PERL script to select only uniquely aligned read pairs (BWA mapping score ≥1), where one end aligned to the bait region and the other end aligned to other regions. Finally, tags within a 100 bp range were merged as a unique interacting site using BEDTools [36]. Singlet interacting sites were considered as background noise and removed.

For enzyme-based 4C-Seq data, the sequencing reads with 5’ end aligned to the forward inverse PCR primer sequence were selected. The rest part of the selected reads (including HindIII sites) was mapped to the mm9 assembly using BWA to locate ligation sites in the genome. The mapped ligated HindIII sites were further matched to a reduced genome with the locations of all HindIII sites included.

### Statistical model to identify enriched interacting regions for sonication-based 4C-Seq data

A statistical model was built to identify interacting regions that exhibit a higher frequency of interactions with the bait than expected from random collision of the chromosomes [18]. For every interacting site *i* on chromosome W (length L_W_), the number of interacting sites within a certain window *w* with length l_W_ (±1 Mb for inter-chromosomal interactions and ±200 kb for intra-chromosomal interactions in our study) from the analyzed site was counted as C_*i,w*_, and a z-score was calculated as an enrichment score:

$$z_{i}=\frac{\left(C_{i\;w}-\mu_{W}\right)}{\sqrt{\mu_{W}\;\left(1-P_{W}\right)}}$$

in which p_W_ = μ_W_/l_W_ (μ_W_ is the expected number of interacting sites in window *w* on chromosome W).

Statistical significance was further assigned to each interacting site by a FDR-based approach. Briefly, we randomly permutated calculated z-score data for every chromosome 100 times, and chose interacting sites with a false discovery rate (FDR) ≤ 5% or FDR ≤ 20% as positively interacting sites for inter-chromosomal or intra-chromosomal interactions. FDR for each site was calculated by counting the number of randomly permutated Z-scores that are above experimentally determined Z-score. All the interacting sites within ±1 Mb range of a positively interacting site were clustered as an enriched interacting domain. Overlapping interacting domains were further merged together.

### ChIP-Seq data

The ChIP-Seq peak data of histone variants for mouse ES cells were retrieved from the ENCODE database (http://genome.ucsc.edu/ENCODE). The ChIP-Seq raw read files of 15 DNA-binding proteins were downloaded from the GEO database (accession number GSE11431).

### Statistical analysis

Statistical analysis was executed and plotted using the R software suite (http://www.r-project.org/). The conversion of genomic coordinates between different genome assemblies was done by liftover software tool (http://www.genome.ucsc.edu/).

## Availability of supporting data

The sequencing data has been deposited to the GEO database (accession number GSE43776, GSE45418).

## Competing interest

All authors declare that they have no competing interest.

## Authors’ contributions

FG, WL and KW designed the project. FG performed the research and implemented the pipeline. ZW prepared the enzyme-based 4C library samples for sequencing. FG, WL and KW wrote the paper. All authors read and approved the final manuscripts.

## Supplementary Material

Additional file 1 Summary of mapping results of 4C-Seq data. Click here for file

Additional file 2: Figure S1 Distribution of read counts at all inter-chromosomal interaction sites. Data generated from the biological replicates BR1 and BR2 were compared. Both enzyme-based and sonication-based data were included in the plots. Click here for file

Additional file 3: Figure S2 Reproducibility of inter-chromosomal interactions at different resolutions. Density scatter plots of inter-chromosomal interactions between the biological replicate data at resolutions of (A),1 Mb for sonication-based data; (B), 250 6-bp cutter sites for enzyme-based data; (C), 500 kb for sonication-based data; (D), 125 6-bp cutter sites for enzyme-based data. Click here for file
