# Supplementary material for: Comparative analysis of 4C-Seq data generated from enzyme-based and sonication-based methods
Source: BMC Genomics. 2013 May 24;14:345. doi: 10.1186/1471-2164-14-345 (PMC3679908; doi:10.1186/1471-2164-14-345)
Supplement: Additional file 1 — Summary of mapping results of 4C-Seq data. [file 1471-2164-14-345-S1.doc]

**Table S1. Mapping of sonication-based next-generation sequencing data**

Library Name: Sonicate-BR1

| Total 91-bp reads: 24,703,628 (100%) | | | | |  |
| --- | --- | --- | --- | --- | --- |
| Mapped reads | | | Unmapped reads | |  |
| 19,150,410 (77.52%) | | | 5,553,218 (22.48%) | |  |
| Uniquely mapped | | Non-uniquely mapped |  | |  |
| 19,136,196 (77.46%) | | 14,214 (0.06%) |  |  | |
| Mapped to bait locus | Mapped to distal regions |  |  |  | |
| 19,029,454 (77.03%) | 106,742 (0.43%) |  |  |  | |

Library Name: Sonicate-BR2

| Total 91-bp reads: 24,192,856 (100%) | | | | |  |
| --- | --- | --- | --- | --- | --- |
| Mapped reads | | | Unmapped reads | |  |
| 18,580,224 (76.80%) | | | 5,612,632 (23.20%) | |  |
| Uniquely mapped | | Non-uniquely mapped |  | |  |
| 18,575,180 (76.78%) | | 5,044 (0.02%) |  |  | |
| Mapped to bait locus | Mapped to distal regions |  |  |  | |
| 18,542,445 (76.64%) | 32,735 (0.14%) |  |  |  | |

**Table S2. Summary of end-tag mapping results for sonication-based 4C-Seq data**

| Library Name | Total junction tag pairs | Proximal junction tag pairs^+^ | Distal junction tag pairs* | Unique distal intra- sites | Unique distal inter-sites |
| --- | --- | --- | --- | --- | --- |
| Sonication-BR1 | 2,427,459 | 2,171,834 | 255,625 | 214 | 5515 |
| Sonication-BR2 | 2,922,907 | 2,720,132 | 202,775 | 165 | 4238 |

^+^Proximal junctions include intra-chromosomal interactions less than 10kb from the bait.

*Distal junctions include intra-chromosomal interactions not less than 10kb from the bait and inter-chromosomal interactions.

**Table S3. Summary of mapping results for enzyme-based 4C-Seq data**

| Library Name | Total junction tags (HindIII) | Proximal junction tags (HindIII)* | Distal junction tags (HindIII)* | Unique distal intra-sites | Unique distal inter-sites |
| --- | --- | --- | --- | --- | --- |
| Enzyme-BR1 | 7,472,712 | 5,016,047 | 2,456,665 | 3,008 | 3,459 |
| Enzyme-BR2 | 4,790,945 | 3,007,358 | 1,783,587 | 1,985 | 2,245 |

^+^Proximal junctions include intra-chromosomal interactions less than 1Mb from the bait.

*Distal junctions include intra-chromosomal interactions not less than 1Mb from the bait and inter-chromosomal interactions.
