# Supplementary figures and images for: Comparative analysis of 4C-Seq data generated from enzyme-based and sonication-based methods
Source: BMC Genomics. 2013 May 24;14:345. doi: 10.1186/1471-2164-14-345 (PMC3679908; doi:10.1186/1471-2164-14-345)

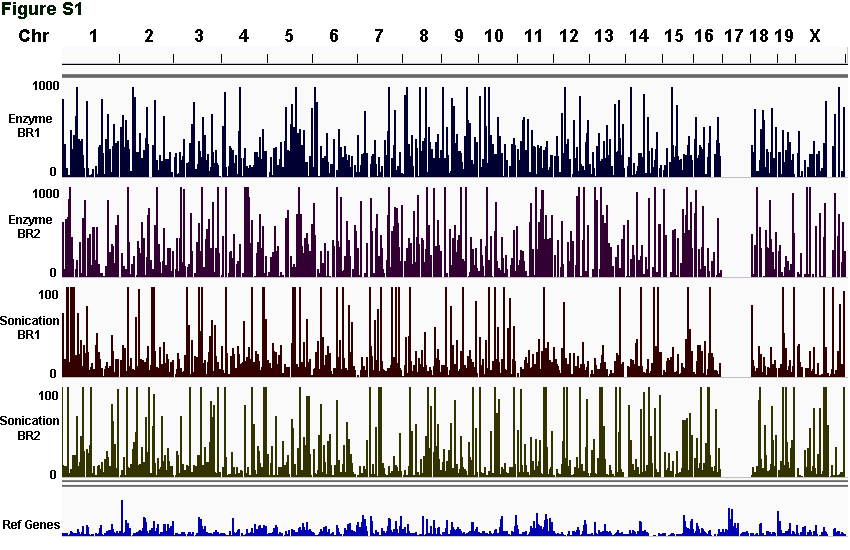

Supplement: Additional file 2: Figure S1 — Distribution of read counts at all inter-chromosomal interaction sites. Data generated from the biological replicates BR1 and BR2 were compared. Both enzyme-based and sonication-based data were included in the plots. [file 1471-2164-14-345-S2.tiff]

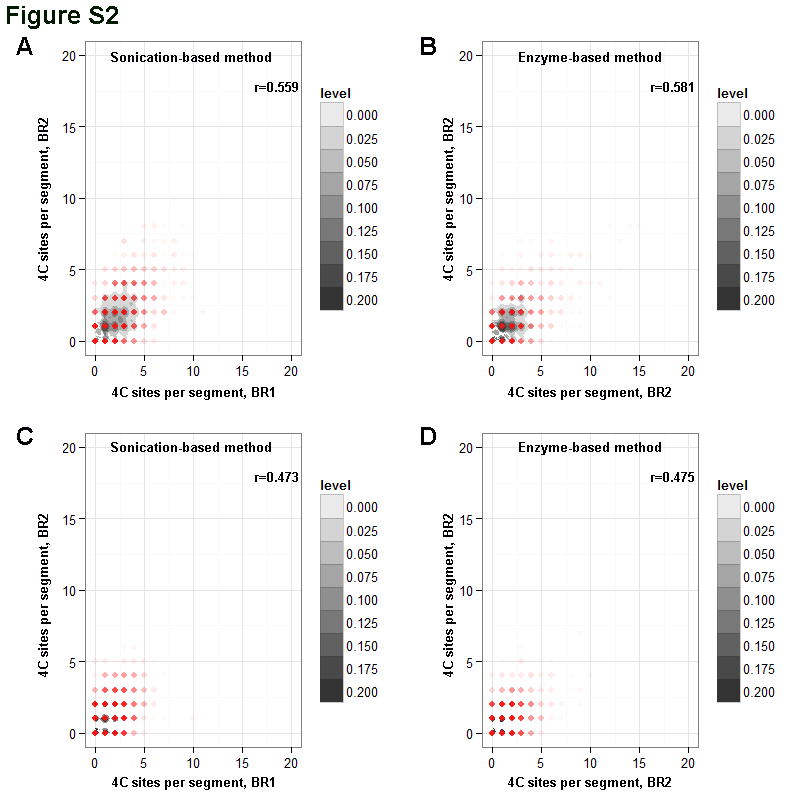

Supplement: Additional file 3: Figure S2 — Reproducibility of inter-chromosomal interactions at different resolutions. Density scatter plots of inter-chromosomal interactions between the biological replicate data at resolutions of (A),1 Mb for sonication-based data; (B), 250 6-bp cutter sites for enzyme-based data; (C), 500 kb for sonication-based data; (D), 125 6-bp cutter sites for enzyme-based data. [file 1471-2164-14-345-S3.tiff]
